# Dexamethasone Improves Cardiovascular Outcomes in Critically Ill COVID-19, a Real World Scenario Multicenter Analysis

**DOI:** 10.3389/fmed.2022.808221

**Published:** 2022-02-02

**Authors:** Peter Jirak, Vincent van Almsick, Dimitrios Dimitroulis, Moritz Mirna, Clemens Seelmaier, Zornitsa Shomanova, Bernhard Wernly, Dilvin Semo, Daniel Dankl, Magdalena Mahringer, Michael Lichtenauer, Uta C. Hoppe, Holger Reinecke, Rudin Pistulli, Robert Larbig, Lukas J. Motloch

**Affiliations:** ^1^Clinic II for Internal Medicine, University Hospital Salzburg, Paracelsus Medical University, Salzburg, Austria; ^2^Department of Cardiology I - Coronary and Peripheral Vascular Disease, Heart Failure, University Hospital Münster, Münster, Germany; ^3^Division of Cardiology, Hospital Maria Hilf Mönchengladbach, Mönchengladbach, Germany; ^4^Department of Anesthesiology, Perioperative Care, and Intensive Care Medicine, University Hospital Salzburg, Paracelsus Medical University, Salzburg, Austria; ^5^Center for Public Health and Healthcare Research, Paracelsus Medical University, Salzburg, Austria; ^6^Department of Internal Medicine, General Hospital Oberndorf, Teaching Hospital of the Paracelsus Medical University, Salzburg, Austria; ^7^Department of Cardiology II - Electrophysiology, University Hospital Münster, Münster, Germany

**Keywords:** cardiac injury, COVID-19, dexamethasone, pulmonary embolism, anticoagulation

## Abstract

**Background:**

Severe COVID-19 pneumonia requiring intensive care treatment remains a clinical challenge to date. Dexamethasone was reported as a promising treatment option, leading to a reduction of mortality rates in severe COVID-19 disease. However, the effect of dexamethasone treatment on cardiac injury and pulmonary embolism remains largely elusive.

**Methods:**

In total 178 critically ill COVID-19 patients requiring intensive care treatment and mechanical ventilation were recruited in three European medical centres and included in the present retrospective study. One hundred thirteen patients (63.5%) were treated with dexamethasone for a median duration of 10 days (IQR 9–10). Sixty five patients (36.5%) constituted the non-dexamethasone control group.

**Results:**

While peak inflammatory markers were reduced by dexamethasone treatment, the therapy also led to a significant reduction in peak troponin levels (231 vs. 700% indicated as relative to cut off value, *p* = 0.001). Similar, dexamethasone resulted in significantly decreased peak D-Dimer levels (2.16 mg/l vs. 6.14 mg/l, *p* = 0.002) reflected by a significant reduction in pulmonary embolism rate (4.4 vs. 20.0%, *p* = 0.001). The antithrombotic effect of dexamethasone treatment was also evident in the presence of therapeutic anticoagulation (pulmonary embolism rate: 6 vs. 34.4%, *p* < 0.001). Of note, no significant changes in baseline characteristics were observed between the dexamethasone and non-dexamethasone group.

**Conclusion:**

In severe COVID-19, anti-inflammatory effects of dexamethasone treatment seem to be associated with a significant reduction in myocardial injury. Similar, a significant decrease in pulmonary embolism, independent of anticoagulation, was evident, emphasizing the beneficial effect of dexamethasone treatment in severe COVID-19.

## Introduction

Acute Respiratory Distress Syndrome (ARDS) caused by coronavirus disease 2019 (COVID-19) remains one of the major challenges in current intensive care treatment worldwide (1). In addition to acute hypoxemia with all its known consequences, the impact of COVID-19 on different organ systems such as the cardiovascular system was repeatedly discussed (2). Still, the influence of COVID-19 on the cardiovascular system is not fully clarified yet (3). First studies have proposed high rates of myocardial injury in COVID-19 infection (4, 5). Similar, acute myocardial damage has emerged as an independent risk factor for a severe course of COVID-19 disease (2, 6). However, according to recent studies, cardiac involvement may not represent a specific feature of COVID-19 but is more likely the result of a high inflammatory burden in the context of a severe disease process (7). Accordingly, a similar effect of COVID-19 on cardiac injury compared to severe non-COVID pneumonia was reported (8, 9). In addition to cardiac injury, an increased thrombogenicity resulting in high rates of pulmonary embolism and other thromboembolic events has been observed in COVID-19 (10, 11). Interestingly, the effect of anticoagulation might be attenuated in the context of COVID-19. While first single-centre studies proposed a beneficial effect of therapeutic anticoagulation on survival rates, randomized multicenter studies failed to demonstrate a reduction in mortality in COVID-19 patients receiving therapeutic anticoagulation (12–16).

At the beginning of the first wave of the pandemic, an international consensus on the treatment of severe COVID-19 was lacking. Additionally, an overstrain of health-care systems worldwide further complicated medical treatment (17, 18). Accordingly, various and in part experimental antiviral therapeutic approaches were subject to clinical investigations during the last year. On this regard, various therapies including remdesivir (19), favipiravir (20) or mavrilimumab (21) could not demonstrate a significant survival benefit in the context of severe COVID-19 disease.

In contrast, dexamethasone therapy was reported to significantly reduce mortality rates in the RECOVERY trial (22). Treatment with dexamethasone resulted in a reduced mortality at 28-days in patients suffering from severe COVID-19 disease, requiring oxygen support or mechanical ventilation (22). Similarly, in the CoDEX trial the number of ventilator-free days over 28 days was shown to decrease significantly due to dexamethasone treatment (23). However, while dexamethasone treatment seems to have a beneficial impact especially in severe COVID-19, the effects of this treatment regimen on myocardial injury and thrombotic events remain largely unknown. Accordingly, in this study we aimed to investigate the effects of dexamethasone treatment on cardiac injury and thromboembolic events in severe COVID-19 disease requiring mechanical ventilation. Since both processes are promoted by a COVID-19 dependent high inflammatory burden (12), we hypothesized that dexamethasone treatment might result in a reduction of myocardial injury as well as thrombotic events.

## Methods

The present study was conducted in three tertiary centers in Germany and Austria in accordance with standards of good clinical practice and the principles of the Declaration of Helsinki. The study was approved by the respective local ethic committees (University Hospital Münster Nr. 2020-306-f-S, Maria Hilf Hospital Mönchengladbach: Nr. 143/2020, and University Hospital Salzburg: Nr. 1071/2020).

### Study Cohorts

For this study, we retrospectively screened 224 consecutive COVID-19 patients requiring intensive care treatment and mechanical ventilation (defined as requirement for high flow nasal cannula, non-invasive and/or invasive ventilation) due to critical illness treated between 4/2020 and 1/2021. To avoid bias effects of further applied COVID-19 specific medications including tocilizumab, remdesivir and sarilumab, 46 patients treated with at least one of these substances were excluded from further analyses. Further investigations were therefore conducted in 178 critically ill patients treated due to COVID-19 with only dexamethasone or no further specific pharmacological therapies for COVID-19. Patients not receiving dexamethasone were treated during the first period of the pandemic, before recommendation for dexamethasone therapy. In the patients receiving dexamethasone, therapy was initiated (6 mg once a day) at the time point when oxygen therapy was necessary independently of the requirement for intensive care treatment. In our cohort, this standard procedure was started in July 2020, after the publication of the RECOVERY trial results (22). Intensive care treatment was initiated if patients required further respiratory support (at least nasal high flow cannula) or were hemodynamically unstable.

The diagnosis of COVID-19 was established in the presence of a positive result of oropharyngeal or/and nasopharyngeal swabs test shown by real-time reverse transcription–polymerase chain reaction assay for COVID-19 (performed according to the manufacturer). Additionally, the presence of a chest radiography and/or computertomography of the thorax indicative for COVID-19-related pneumonia according to current recommendations was required to confirm the diagnosis of COVID-19. Intensive care treatment was concluded in all patients included in the study, meaning they either were discharged or had died at the time of data analysis.

### Data Collection

In all eligible patients, patient data were obtained during their intensive care treatment. Demographics, medical history, laboratory examinations, comorbidities, complications, specific treatment measures, and outcomes were collected and analyzed. To account for the usage of different hs-Tn assays in the recruiting centres, levels of hs-Tn are given as the relative value (%) of the troponin assay-specific cut-off value (14.0 ng/L for hs-TnT and 51.4 ng/mL for hs-TnI). Further diagnostic follow-up for stroke or pulmonary embolism was initiated dependent on patient's clinic, according to physician guided decision making. Diagnoses of pulmonary embolism and stroke were established and treated according to current guidelines (24, 25). If available (bedside) transthoracic echocardiography (TTE) obtained at the first day after admission to the intensive care unit was investigated. TTE and analyses of relevant findings were performed by an experienced cardiologist or an experienced intensive care physician. Left ventricular ejection fraction (LVEF) was estimated using eyeballing by an experienced cardiologist or an experienced intensive care physician. Diagnosis of reduced LVEF was assumed, if echocardiography at admission to the intensive care ward revealed an LVEF <50%.

### Statistical Analysis

The statistical analysis was carried out blindly by our statistical analytic team using SPSS (Version 22.0, SPSS Inc., USA) and GraphPad Prism (GraphPad Software, USA). Data distribution, skew and kurtosis were assessed visually and by conducting a Kolmogorov-Smirnov test prior to analysis. Since data were not normally distributed, median ± interquartile-range (IQR) were depicted in the manuscript. Medians were compared by applying a Mann-Whitney *U*-Test, whereas categorical data between groups were assessed using Fisher's exact test. Bonferroni-Holm correction was performed to adjust for alpha inflation. A *p*-value of <0.05 was considered statistically significant.

## Results

### Baseline Characteristics

In total, 178 patients were enrolled in this study. Of these, 113 patients (63.5%) were treated with dexamethasone for a median duration of 10 days at the intensive care ward (IQR 9–10). Sixty five patients (36.5%) constituted the control group. Baseline characteristics of enrolled patients are depicted in Table 1. In the total study cohort, the median age was 66 years (IQR 57–77) and the majority of patients were male (72.5%, *n* = 129). We observed no statistically significant differences in baseline characteristics or comorbidities between the two groups investigated (see Table 1).

**Table 1:** Baseline characteristics and comorbidities of patients within the two subgroups.

	**Dexamethasone (*****n*** **= 113)**		**No dexamethasone (*****n*** **= 65)**		
**Baseline characteristics**	**Median**	**IQR**	**Median**	**IQR**	***P*-value**
Age (years)	66	59–78	64	56–76	0.313
BMI (kg/m^2^)	29	26–33	27	25–31	0.099
	**%**	***N***	**%**	***N***	***P*** **-value**
Male sex	72.6	82	72.3	47	0.970
Diabetes mellitus	36.3	41	29.2	19	0.411
Arterial hypertension	66.4	75	53.8	35	0.111
History of smoking	29.2	33	21.5	14	0.294
Coronary artery disease	20.4	23	16.9	11	0.693
Peripheral artery disease	6.2	7	6.2	4	0.991
Atrial fibrillation	17.7	20	10.8	7	0.279
Heart failure	15.0	17	9.2	6	0.355
Obstructive lung disease	23.0	26	13.8	9	0.172
Structural lung disease	8.0	9	4.6	3	0.540
Malignancy	7.1	8	9.2	6	0.773
History of thromboembolism	12.4	14	6.2	4	0.209
Therapeutic anticoagulation during ICU stay	74.3	84	49.2	32	0.001

*BMI, body mass index; IQR, interquartile range*.

### Parameters of Cardiac Function

**Figure 1:**
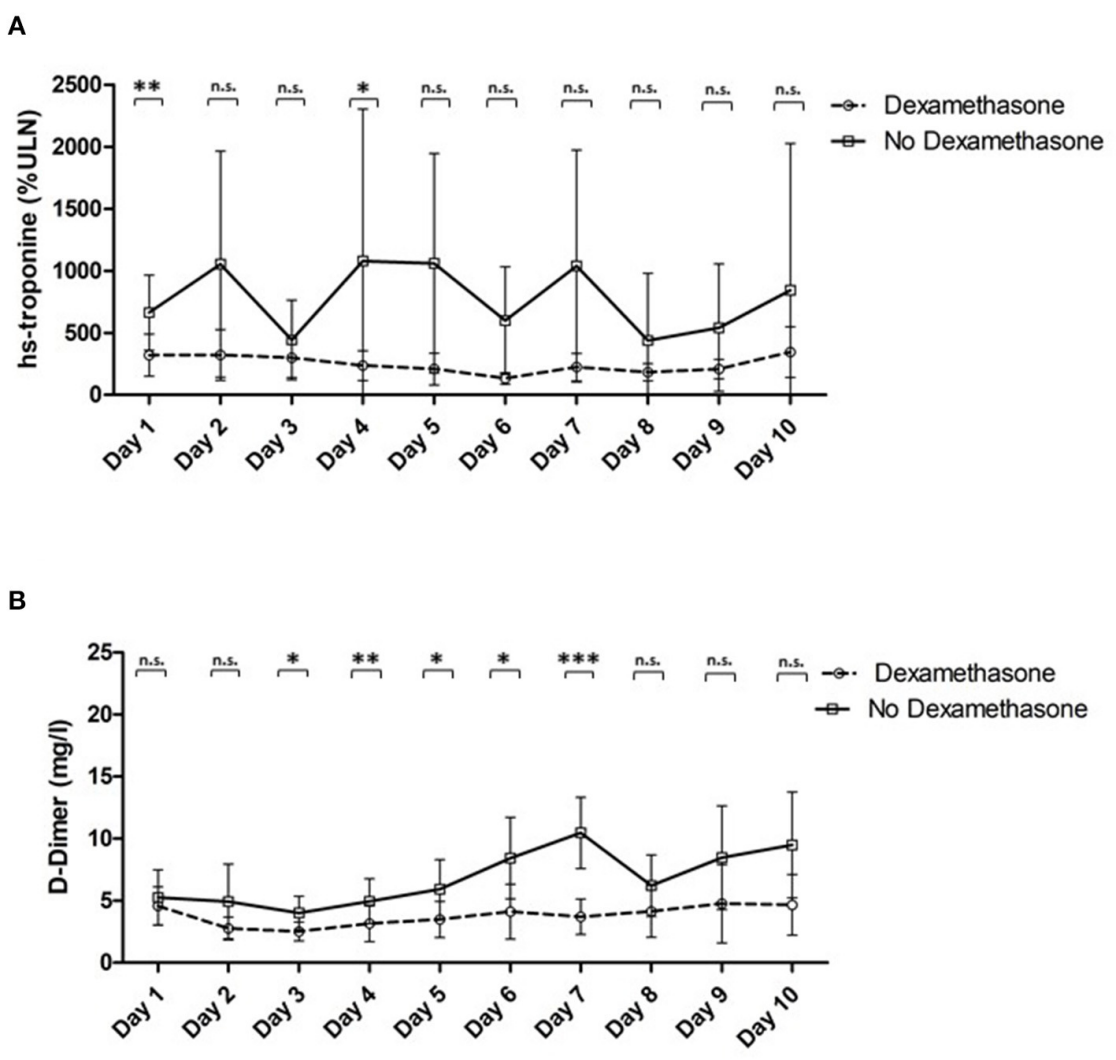
**(A)** plasma levels of high sensitivity troponine normalized to %ULN in patients treated with dexamethasone vs. controls, **(B)** plasma levels of D-dimer in patients treated with dexamethasone vs. controls. *indicates a *p* of <0.05; **a *p* of <0.01 and *** a *p* of <0.001; n.s., not significant. hs, high sensitivity; ULN, upper limit of norm.

Throughout the study, patients treated with dexamethasone showed significantly reduced plasma levels of high sensitivity troponin [troponin max.: median: 231% (IQR 89–571) vs. 700% (IQR 164–2,216), *p* = 0.001; see Table 2; Figure 1A] despite no statistically significant differences in LV systolic function at admission. Similar, a trend toward lower NT-proBNP levels was evident [1,224 pg/ml (IQR 318–3,375) vs. 2,829 pg/ml (IQR 484–9,372), *p* = 0.065; see Table 2].

**Table 2:** Parameters of cardiac function and cardiac laboratory parameters of patients treated with dexamethasone vs. controls.

	**Dexamethasone (*****n*** **= 113)**		**No dexamethasone (*****n*** **= 65)**		
**Echocardiography**	**%**	***N***	**%**	***N***	***P*-value**
LV systolic function					0.304
Normal	73.1	68	81.3	39	0.924^*^
Mildly reduced	14.0	13	4.2	2	0.352^*^
Moderately reduced	7.5	7	6.3	3	0.990^*^
Severly reduced	5.4	5	8.3	4	0.990^*^
LV dilated	8.9	8	6.3	3	0.747
RV dilated	12.2	11	12.8	6	0.927
Pericardial effusion	2.2	2	8.5	4	0.179
**Cardiac laboratory parameters**	**Median**	**IQR**	**Median**	**IQR**	***P*** **-value**
High sensitivity troponine max (% ULN)	231	89–571	700	164–2,216	0.001
CK max (U/L)	338	142–756	357	170–892	0.508
CK-MB max (U/L)	31	23–52	30	22–44	0.604
pBNP max (pg/ml)	1,224	318–3,375	2,829	484–9,372	0.065

* *indicates p-value after Bonferroni-Holm correction for multiple testing. CK, creatinine kinase; CK-MB, creatinine kinase muscle-brain type; IQR, interquartile range; LV, left ventricle/ventricular; pBNP, pro brain natriuretic peptide; RV, right ventricle*.

### Other Laboratory Parameters

Dexamethasone resulted in a significant attenuation of the peak plasma levels of D-Dimer [D-Dimer max.: median: 2.16 mg/l (IQR 0.94–5.16) vs. 6.14 mg/l (IQR 1.78–16.48), *p* = 0.002; see Table 3; Figure 1B]. Furthermore, we observed a significant amelioration of CRP and a trend toward a decrease of interleukin 6 levels [CRP max.: median: 20 ng/mL (IQR 12–28) vs. 22 ng/mL (IQR 14–37), *p* = 0.043; IL-6 max.: median: 192 pg/mL (IQR 78–533) vs. 708 pg/mL (550–885), *p* = 0.085; see Table 3].

**Table 3:** Other relevant laboratory parameters in patients of both investigated groups.

	**Dexamethasone (*****n*** **= 113)**		**No dexamethasone (*****n*** **= 65)**		
**Laboratory parameters**	**Median**	**IQR**	**Median**	**IQR**	***P*-value**
Lactate max (U/L)	2.78	2.10–3.96	2.60	1.80–4.85	0.729
pH min	7.27	7.15–7.38	7.24	7.13–7.37	0.523
Creatinine max (mg/dL)	1.44	0.97–2.75	1.99	1.06–3.50	0.188
Potassium min (mmol/l)	3.52	3.30–4.00	3.60	3.33–3.90	0.920
Leukocyte count max (G/L)	15	11–20	15	11–23	0.462
Lymphocyte min (G/L)	4.9	1.0–13.0	4.4	0.9–13.9	0.973
D-Dimer max (mg/l)	2.16	0.94–5.16	6.14	1.78–16.48	0.002
CRP max (ng/mL)	20	12–28	22	14–37	0.043
PCT max (ng/mL)	1.00	0.20–3.04	1.88	0.50–7.60	0.086
Interleukin 6 max (pg/mL)	192	78–533	377	762	0.085
Fibrinogen max (mg/dL)	696	483–799	708	550–885	0.634

#### Outcomes and Required ICU Therapy

**Figure 2:**
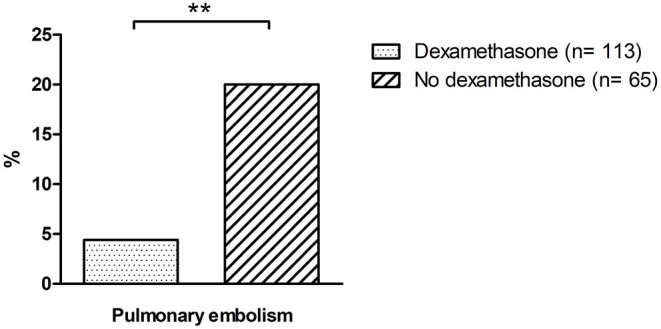
Incidence of pulmonary embolism in patients treated with dexamethasone vs. controls. * indicates a *p* of <0.05; ** a *p* of <0.01 and *** a *p* of <0.001; n.s., not significant.

We observed no statistically significant differences in intensive care mortality as well as the incidence of stroke, ventricular arrhythmias or LV systolic dysfunction between patients of both groups (see Table 4). However, dexamethasone resulted in a significant attenuation of the rate of pulmonary embolism (4.4 vs. 20%, *p* = 0.001; see Table 4; Figure 2) in patients of the treatment group. Furthermore, we observed a trend toward a reduced duration of ICU stays in patients treated with dexamethasone, which, however, remained statistically insignificant in our cohort [median: 10 days (IQR 6–16) vs. 13 days (IQR 6–28), *p* = 0.055; see Table 4].

**Table 4:** Required ICU therapies and outcomes in patients treated with dexamethasone vs. controls.

	**Dexamethasone (*****n*** **= 113)**		**No dexamethasone (*****n*** **= 65)**		
**Required ICU therapies and outcomes**	**%**	***N***	**%**	***N***	***P*-value**
ICU mortality	44.2	50	39.3	24	0.630
**Required ICU therapy**					
ECMO	21.2	24	15.4	10	0.429
Hemofiltration	20.4	23	27.7	18	0.273
Catecholamines	69.0	78	67.7	44	0.915
Electrical cardioversion	3.5	4	7.7	5	0.334
**Complications**					
CPR	3.6	4	3.1	2	0.853
Bleeding	1.8	2	7.8	5	0.102
Pulmonary embolism (PE)	4.4	5	20.0	13	0.001
PE despite therapeutic anticoagulation	6.0	5	34.4	11	<0.0001
Acute cardiac injury	80.9	76	67.4	31	0.092
Stroke	2.7	3	7.7	5	0.143
Deep vein thrombosis	6.2	7	6.7	4	0.760
Ventricular arrhythmia	5.5	6	1.5	1	0.371
	**Median**	**IQR**	**Median**	**IQR**	
Duration of ICU stay (days)	10	6–16	13	6–28	0.055
Duration of invasive ventilation (days)	8	1–14	8	3–18	0.319

*CPR, cardiopulmonary resuscitation; ECMO, extracorporeal membrane oxygenation; BMI, body mass index, IQR, interquartile range*.

### Anticoagulation

**Figure 3:**
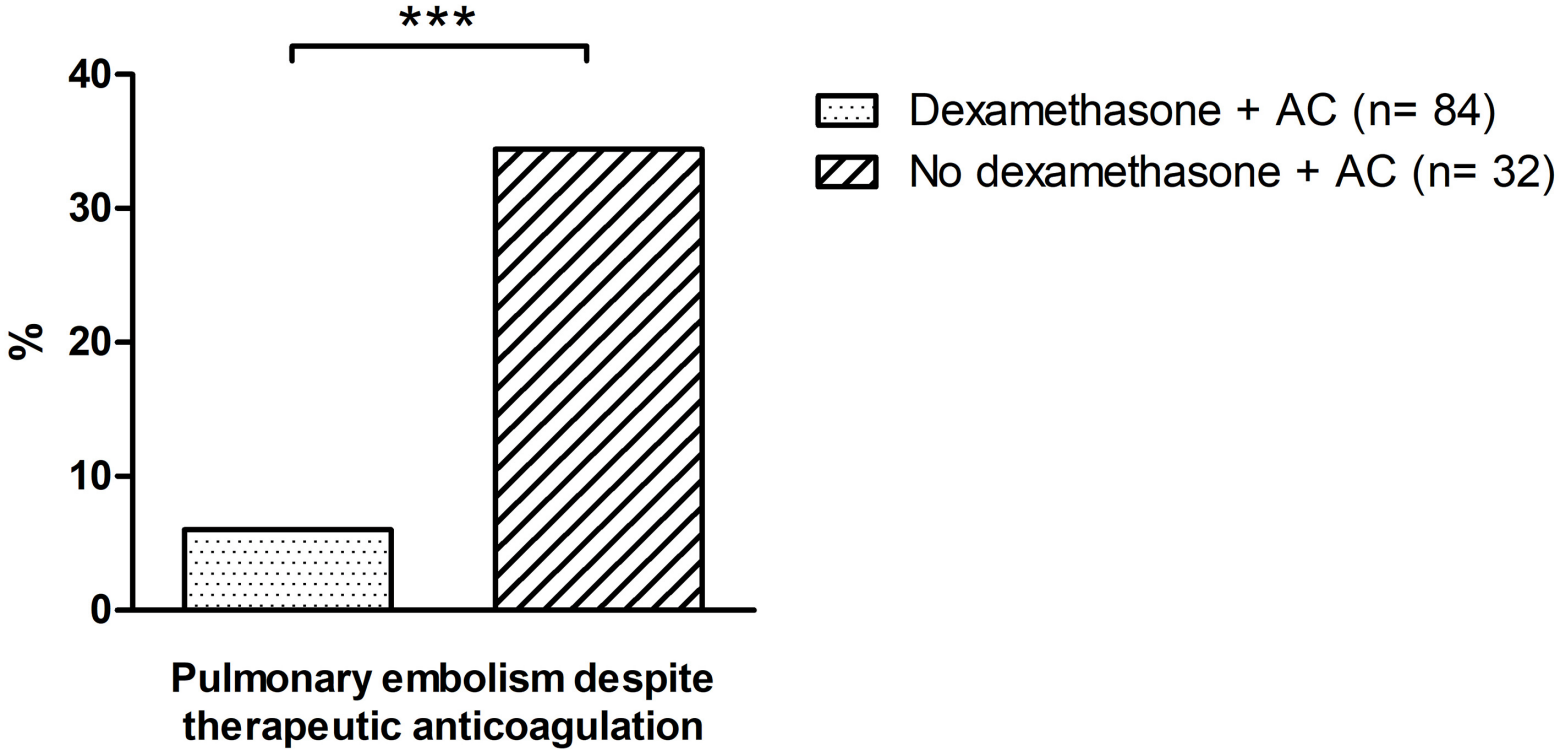
Incidence of pulmonary embolism despite therapeutic anticoagulation in patients treated with dexamethasone vs. controls. *indicates a *p* of <0.05; ** a *p* of <0.01 and *** a *p* of <0.001, n.s., not significant. AC, anticoagulation.

In the dexamethasone group, 84 patients (7.3%) received therapeutic and 29 patients (25.7%) received prophylactic anticoagulation. In the non-dexamethasone group, 32 patients (49.2%) received therapeutic and 31 patients (50.8%) received prophylactic anticoagulation. In patients receiving prophylactic anticoagulation, no pulmonary embolism was detected in the dexamethasone group, while two patients in the non-dexamethasone group developed pulmonary embolism. Similar, also in the presence of therapeutic anticoagulation, a protective effect of dexamethasone was evident. While five patients (6%) suffered pulmonary embolism in the dexamethasone group, 11 patients (34.4%) in the non-dexamethasone group had pulmonary embolism (*p* = 0.001) (Figure 3). Baseline characteristics and thromboembolic risk factors of patients on therapeutic and prophylactic anticoagulation are depicted in Supplementary Table 1.

## Discussion

Severe COVID-19 disease remains an ongoing global health challenge. Along with acute lung injury resulting in ARDS, also a high burden of myocardial damage was reported as a frequent finding (26, 27). On this regard, the high inflammatory burden in severe COVID-19 disease constitutes a major promoting factor. However, the ideal treatment regimen in these patients remains debatable. In the RECOVERY trial and the CoDEX trial, dexamethasone therapy resulted in a reduced mortality at and a higher number of ventilator-free days over 28 days (22, 23). To further elucidate the mechanisms behind these beneficial effects, we aimed to investigate the effects of dexamethasone treatment in severe COVID-19 on cardiac injury and thrombotic events in a real-world scenario.

Regarding baseline characteristics, we did not observe significant differences between the dexamethasone and non-dexamethasone group for age, sex and comorbidities. However, patients in the dexamethasone group showed a tendency toward a higher BMI. Given the lack of significant differences in the baseline characteristics, no relevant impact on our results is assumed on this regard.

However, inflammatory parameters seemed to display significant changes between the two groups. A significant decrease in levels of maximum CRP and a trend toward reduced Interleukin-6 levels were revealed in the dexamethasone group. Accordingly, this finding might reflect a potential impact of dexamethasone therapy on inflammatory activity in our cohort.

Matching our expectations, we found troponin levels to be significantly reduced in the dexamethasone group during the first 10 days of intensive care treatment, indicating a beneficial effect of dexamethasone treatment on cardiac injury. Similar to former studies, troponin was used as surrogate parameter for cardiac dysfunction and cardiac injury on this regard (28, 29). Cardiac injury can be promoted by different factors in the context of severe infection (28–30). These comprise inflammatory damage leading to pericarditis and myocarditis as well as an exacerbation of heart failure or coronary artery disease (28–30). Accordingly, in addition to their reflection of cardiac injury, troponin levels were also reported to be of prognostic relevance in the presence of sepsis (28–30). Additionally, recent studies demonstrated an increased risk for cardiac complications and fatal outcomes in the presence of elevated troponin levels in influenza patients, with a significant impact on 30-day mortality rates (31, 32). In the present study, a significant difference in troponin levels was already evident at the first day after admittance to ICU. We speculate that differences in Troponin levels at day 1 may be due to the effect of early dexamethasone treatment before admittance to the ICU. However, cardiac left ventricular ejection fraction obtained by echocardiography was not different between both groups. Still, according to previous data, this parameter does not seem to be specifically affected by severe COVID-19 disease (8). Furthermore, while echocardiography was only performed at the first day after admission, beneficial effects during follow up or at the end of the dexamethasone treatment cannot be excluded by our results. On the other hand, a trend toward a higher rate of pericardial effusion was observed in the non-dexamethasone group, which might constitute the effect of an increased inflammatory burden in this patient cohort (Table 2).

As troponin levels are strongly associated with D-Dimer concentrations in COVID-19 (12), we further investigated this observation. So far, only one study investigated D-Dimer levels in COVID-19 patients treated by steroids (33). Of note, while no changes in D-Dimer levels were observed by the authors, one has to consider that in the mentioned study the authors applied a modified methylprednisolone regime at a late stage of disease (7–10 days after symptoms onset) (33). In contrast to this trial, our real world scenario showed a significant reduction in D-Dimer levels in the dexamethasone group. As D-Dimer concentrations are correlated with inflammatory activity and also prognosis, this finding further emphasizes the potential beneficial effects of dexamethasone treatment in COVID-19 (13).

Correlating with the significant reduction of D-Dimer levels, a significantly lower rate of pulmonary embolisms in patients treated with dexamethasone was observed. Contrary, rates of stroke and deep vein thrombosis (TVT) remained unaltered. This finding might be attributed to a more fulminant clinical presentation of pulmonary embolism in the ICU setting along with a lack of systematic screening for TVT and stroke in our patient collective. Accordingly, a potential underdiagnosis of TVT and stroke must be considered on this regard. Additionally, different rates of anticoagulation must be taken into account, with a significantly higher rate of therapeutic anticoagulation in the dexamethasone cohort. While all patients in our study received at least prophylactic anticoagulation, higher rates of pulmonary embolisms were found in patients receiving therapeutic anticoagulation. While this finding might primarily appear paradox, it must be considered that no definite recommendations on anticoagulation in severe COVID-19 patients exists to date (13). Thus, patients with higher risk for thromboembolic events or higher D-Dimer levels may have been more likely to receive therapeutic anticoagulation. Additionally, patients on therapeutic anticoagulation displayed significantly higher rates of heart failure and atrial fibrillation, indicating a higher cardiovascular disease burden. Of note, the effect of therapeutic anticoagulation seemed at least attenuated in the presence of severe COVID-19 in our patient collective. This is in accordance with recent multicenter studies, where therapeutic anticoagulation failed to provide beneficial effects in critically ill COVID-19 patients and resulted in potential harm in this patient collective (15, 16). Accordingly, this factor must be considered when analyzing these clinically contradicting findings. However, while therapeutic anticoagulation alone did not lead to a reduction of thromboembolism rates, the additive treatment with dexamethasone did result in a significant reduction of pulmonary embolisms on top of therapeutic anticoagulation, emphasizing its antithrombotic effect in COVID-19.

Concerning outcomes, no changes in mortality rates were observed between the two groups. On this regard, the different rates of therapeutic anticoagulation in both cohorts needs to be considered. As therapeutic anticoagulation in the absence of a confirmed PE/DVT was associated with a potential harmful outcome compared to usual-care pharmacologic thromboprophylaxis, anticoagulation rates might be considered a potential confounder (16). Moreover, one must keep in mind that the survival data in our study only accounts for the duration of ICU stay and does not cover a potential delayed survival benefit. Accordingly, our findings do not account for potential medium- and long-term effects of dexamethasone treatment in severe COVID-19 disease. This fact must also be considered with regards to the findings of a reduction of myocardial injury and pulmonary embolism, as these effects could have not only an immediate but also a prolonged impact on survival. With regards to a potential influence of virus mutations on our results, no mutation sequencing was available in our patient collective. However, data obtained for dexamethasone patients covers the time period until 01/2021. The first variants, B.1.1.7 and B.1.351 were declared a variant of concern on 18th of December 2020, followed by P.1 on 11th of January 2021 (34). Thus, given the small time-overlap, a relevant influence of virus mutations on our study results is assumed unlikely.

Nevertheless, to the best of our knowledge, this is the first study showing a major impact of dexamethasone treatment on cardiac injury as well as pulmonary embolism rates, which is a major clinical problem in COVID-19. With respect to limited therapy options for prevention of these malignant thrombotic events, our finding seems to be of high clinical relevance. Of note, while some preliminary studies suggested beneficial effects of therapeutic anticoagulation (35), first randomized trials showed contradictory results (14–16, 36). Based on the current literature, it is assumed that non-critically ill COVID-19 patients might benefit from therapeutic anticoagulation (36, 37) while for critically ill COVID-19 patients, requiring intensive care treatment, no benefits but a potentially harmful effect is observed (15, 16). This data is further supported by our observations, which show a high rate of thrombotic events in anticoagulated patients. Therefore, our results implicate further speculations regarding the potential protective cardiovascular effects of dexamethasone therapy in severe COVID-19. While both cardiac injury and thrombogenicity are associated with COVID-19 related inflammation (8, 12, 38, 39), anti-inflammatory effects seem possible. Furthermore, one could speculate, that a reduction in COVID-19 related vasculitis might further promote protective cardiovascular and antithrombotic effects (40). Nevertheless, this issue is speculative and should be the subject of further COVID-19 related investigations.

## Limitations

The present study has by the retrospective design its limitations, while contributing novel clinical findings. Instead of screening, diagnostic workups for thromboembolic events were only performed when clinically suspected and are therefore probably underestimated. Thus, further confirmation of our findings in a larger study is necessary. While patients not receiving dexamethasone were treated during the first period of the pandemic, limited clinical experience could affect disease management and therefore clinical outcomes. The assessment of cardiac injury was based on cardiac enzymes and echocardiography, while more precise imaging techniques such as MRI including stress testing were not applied. Advanced imaging, as well as coronary and haemodynamic workups, would have provided more information on the ischaemic or inflammatory nature of cardiovascular outcomes. Additionally, there was no structured approach to echocardiographic evaluation and follow-up. Thus, echocardiography was not performed in all patients. More importantly, missing follow-up analyses would have helped to characterize the implications of cardiovascular outcomes in our population. Since our data was investigated in severe COVID-19 requiring mechanical ventilation, our results cannot be applied in less severe COVID-19 clinical scenarios. Accordingly, the hypothesis generating character has to be kept in mind, when interpreting our study results.

## Conclusion

Dexamethasone treatment in severe COVID-19 patients leads to a significant reduction in myocardial injury and pulmonary embolism according to our data. The observed reduction of pulmonary embolisms was independent of rates of anticoagulation, emphasizing the protective effect of dexamethasone treatment in severe COVID-19 disease.

## Data Availability Statement

The raw data supporting the conclusions of this article will be made available by the authors, without undue reservation.

## Ethics Statement

The study was approved by the Respective Local Ethic Committees of the participating Medical Centers: in Germany: University Hospital Münster: Nr. 2020-306-f-S and Maria Hilf Hospital Mönchengladbach: Nr. 143/2020, and in Austria: University Hospital Salzburg: Nr. 1071/2020. Written informed consent for participation was not required for this study in accordance with the national legislation and the institutional requirements.

## Author Contributions

All authors of the manuscript meet the criteria for authorship and contributorship as defined by the ICMJE. All authors contributed to the article and approved the submitted version.

## Conflict of Interest

The authors declare that the research was conducted in the absence of any commercial or financial relationships that could be construed as a potential conflict of interest.

## Publisher's Note

All claims expressed in this article are solely those of the authors and do not necessarily represent those of their affiliated organizations, or those of the publisher, the editors and the reviewers. Any product that may be evaluated in this article, or claim that may be made by its manufacturer, is not guaranteed or endorsed by the publisher.

## Supplementary Material

Supplementary Table 1 Comparison of baseline characteristics and comorbidities of patients receiving therapeutic and prophylactic anticoagulation.
