# Supplementary material for: Dexamethasone Improves Cardiovascular Outcomes in Critically Ill COVID-19, a Real World Scenario Multicenter Analysis
Source: Front Med (Lausanne). 2022 Feb 2;9:808221. doi: 10.3389/fmed.2022.808221 (PMC8847392; doi:10.3389/fmed.2022.808221)
Supplement: Supplementary Table 1 — Comparison of baseline characteristics and comorbidities of patients receiving therapeutic and prophylactic anticoagulation. [file Data_Sheet_1.PDF]

Supplement Table 1

Baseline characteristics	Ther. anticoag. (n= 116)		Proph. anticoag. (n= 62)		
	median	IQR	median	IQR	p-value
Age (years)	66	56-79	66	59-75	0.852
BMI (kg/m²)	29	26-33	29	26-32	0.261
	%	n	%	n	p-value
Male sex	73.3	85	71.0	44	0.860
Diabetes mellitus	35.3	41	30.6	19	0.618
Arterial hypertension	60.3	70	64.5	40	0.630
History of smoking	27.6	32	24.2	15	0.722
Coronary artery disease	21.6	25	14.5	9	0.319
Peripheral artery disease	7.8	9	3.2	2	0.334
Atrial fibrillation	21.6	25	3.2	2	0.002
Heart failure	17.2	20	4.8	3	0.019
Obstructive lung disease	19.8	23	19.4	12	0.940
Structural lung disease	6.9	8	6.5	4	0.910
Malignancy	6.9	8	9.7	6	0.564
History of thromboembolism	12.1	14	6.5	4	0.302
